# Enhanced Asymptomatic Systemic Infection Caused by *Plesiomonas shigelloides* in a Captive Gray Wolf (*Canis lupus*)

**DOI:** 10.3390/vetsci8110280

**Published:** 2021-11-19

**Authors:** Kyoo-Tae Kim, Haeseung Lee, Dongmi Kwak

**Affiliations:** 1 Animal Health Center of Zoo Land, Daejeon O-World Theme Park, Jung-gu, Daejeon 35073, Korea; panvet@koagi.or.kr; 2 Siberian Tiger Conservation Center, Baekdudaegan National Arboretum, Bonghwa-gun, Gyeongbuk 36209, Korea; 3 Department of Veterinary Medicine, College of Veterinary Medicine, Kyungpook National University, Buk-gu, Daegu 41566, Korea; lhs1457@knu.ac.kr; 4 Cardiovascular Research Institute, College of Medicine, Kyungpook National University, Jung-gu, Daegu 41944, Korea

**Keywords:** antimicrobial susceptibility, asymptomatic death, gastroenteric infection, gray wolf, *Plesiomonas shigelloides*

## Abstract

A 7-year-old male gray wolf was found dead at a zoo during exhibition. To determine the cause of death, histological and gross necropsy diagnoses and a molecular analysis were performed. The gross necropsy revealed a swollen abdomen, hemorrhagic exudates around the mouth, splenomegaly, a discolored liver, a congested kidney, hemorrhagic ascites, and dark gray-colored membranes and air bubbles in the fundus of the stomach. Rod-shaped bacteria were found in the liver parenchyma and hemorrhagic ascites using Giemsa staining. The nucleotide sequencing of the cultured bacteria identified the causative agent as *Plesiomonas shigelloides*, which is rarely responsible for systemic infections. This study describes a rare case and the first reported systemic gastrointestinal infection due to *P. shigelloides* in a zoo animal.

## 1. Introduction

*Plesiomonas shigelloides*, a member of the Vibrionaceae family, is a motile, gram-negative, oxidase-positive, facultatively anaerobic bacterium regarded as an opportunistic pathogen in animals and humans [1]. *P. shigelloides* has been reported as pathogenic in fish, reptiles, penguins, foxes, hares, and wolves [1,2,3]. *P. shigelloides* may be found in healthy animals, but it occasionally causes intestinal infection, abscess, ulcerative skin lesions, and septicemia in zoo animals [1,4]. In humans, it causes gastroenteritis and extraintestinal infections, such as septicemia, arthritis, cellulitis, osteomyelitis, and meningitis [5,6,7,8]. Most cases involve fish, as it is rarely found in marine mammals, waterfowl, or zoo animals [1,2,9]. Here, we report a rare case of systemic gastrointestinal infection caused by *P. shigelloides* in a gray wolf (*Canis lupus*) housed in a zoo in Korea.

## 2. Case Presentation

### 2.1. Case Description and Clinical Findings

A 7-year-old male gray wolf, which had been kept in an enclosed facility at the Daejeon O-World Theme Park, located in central Korea (36°17′19.00″ N, 127°23′52.04″ E), was found dead without presenting clinical signs of illness up to that point. The animal was housed for 7 years and was fed with a diet of sanitized commercial uncooked chicken with whole bones.

**Figure 1:**
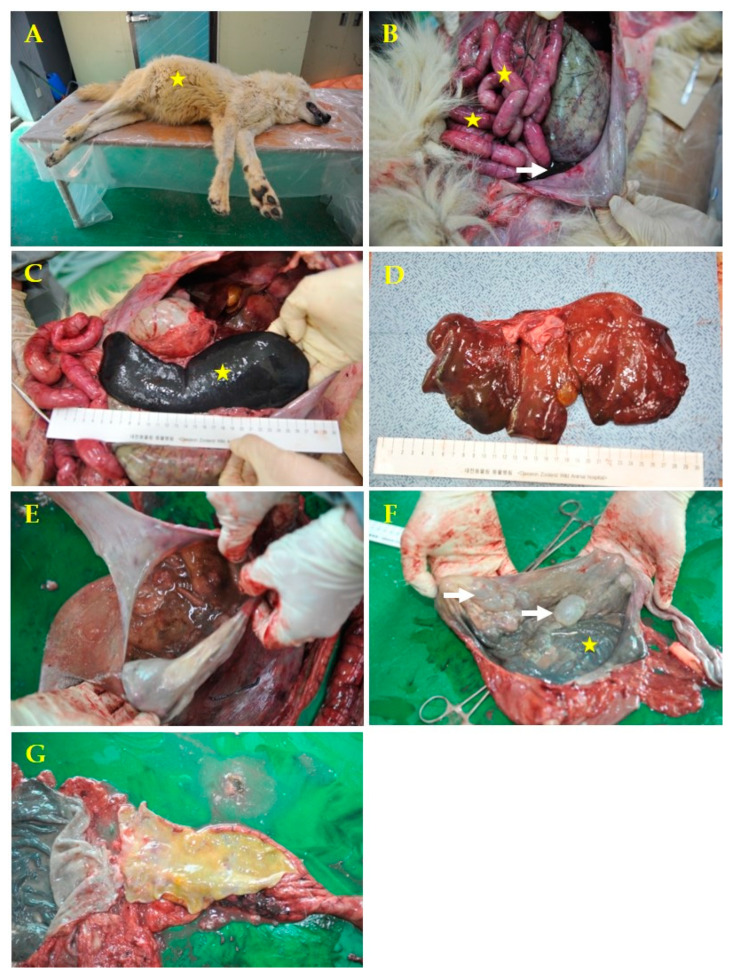
Macroscopic examination of systemic infection caused by *Plesiomonas shigelloides* in a 7-year-old male gray wolf. (**A**) Swollen abdomen (yellow star). (**B**) Swollen gastrointestinal organs, hemorrhagic congestion (yellow stars) with decaying odor, and hemorrhagic ascites (white arrow). (**C**) Severe splenomegaly (yellow star). (**D**) Decrease in the consistency of the parenchyma and mildly diffuse yellowish decolorization of the liver. (**E**) Stomach with ingested food and bad odor. (**F)** The stomach membrane had a dark gray color (yellow star) and several air bubbles in the fundus (white arrows). (**G**) The intestine had yellowish watery contents.

To determine the cause of death, the histological and gross necropsy diagnoses were performed. The necropsy was performed immediately after death, and specimens were collected for laboratory analyses. The carcass presented with a swollen abdomen and hemorrhagic exudates around the mouth (Figure 1A). The necropsy was performed according to standard protocols [10], and gastrointestinal organs were observed to be swollen and hemorrhagically congested, with decaying odor and hemorrhagic ascites (Figure 1B). The spleen was severely swollen (Figure 1C), and the stomach was found to be mixed with semi-liquid exudates and undigested chicken with decaying odor. The mucosal membrane was dark gray, with several air bubbles in the fundus (Figure 1F). In addition, the liver was discolored, the gall bladder was enlarged, and the cortex and medulla of the kidneys were congested (data not shown).

Air bubbles in the gastric mucosa may appear as autolysis bubbles. However, although it is not in the picture, at the time of necropsy the stomach contents were already decomposed and had a bad odor. The air bubbles were not opened, and the contents were identified as air bubbles. The peritoneal membrane was severely congestive. The intestinal contents did not contain blood. The erythema was presumed to be caused by *Plesiomonas* endotoxins; this was confirmed through blood ascites in the abdominal cavity. The liver showed a diffuse mild yellowish fatty appearance.

**Figure 2:**
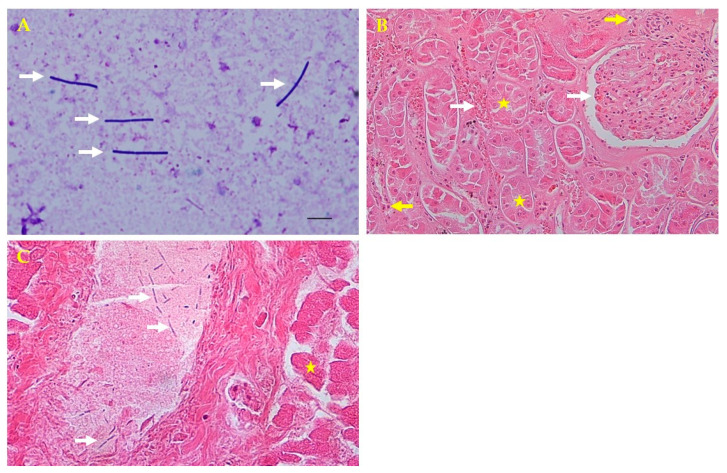
Microscopic and histopathologic findings. (**A**) Direct smear from the liver parenchyma showing rod-shaped bacteria colonies by Giemsa staining (white arrows; 1000×). (**B**) Kidney tubular necrosis (yellow stars) and glomerular and intertubular congestion (white arrows) with inflammatory cells (yellow arrows) by hematoxylin and eosin (H&E) staining (200×). (**C**) Thread-like bacteria (white arrows) in the portal vein and hepatocellular necrosis in the liver (yellow star) by H&E staining (400×).

Samples taken from internal organs (liver, kidneys, spleen, and heart) and hemorrhagic ascites were submitted to the zoo laboratory for bacterial culture and antimicrobial susceptibility tests and to the College of Veterinary Medicine, Kyungpook National University, Daegu, Korea, for molecular diagnosis. The samples were cultured on blood agar (Asan Pharmacy, Seoul, Korea) at 37 °C for 18 h. A pure culture of hemolytic, dew drop colonies was isolated from the agar after the incubation period. Giemsa staining showed gram-negative, coccobacillic, violet-tinted, rod-shaped colonies that were found in the internal organs and ascites upon microscopic examination (Figure 2A). These microorganisms were identified as *P. shigelloides* using a biochemical API 20NE identification kit (bioMérieux, Marcy l’Etoile, France) [9]. A necropsy and an examination of the central nervous system were performed, but no specific external findings were observed, and the causative bacteria were not cultured.

For the histopathologic analysis, tissue samples (liver, kidneys, spleen, and heart) were collected and fixed in 10% neutral buffered formalin for 2 weeks, embedded in paraffin, sectioned at 4 μm, and stained with hematoxylin and eosin for microscopic examination. Histopathologically, tubular necrosis, and glomerular congestion were observed in the kidneys. The interspaces between the renal tubules were found to be infiltrated with blood and inflammatory cells (Figure 2B). The liver portal vein had thread-like bacteria and hepatocellular necrosis (Figure 2C). Although autolysis is not entirely impossible, cells with normal nuclei were observed around some necrotic cells found in kidney tissues. Inflammatory cells were also observed around the tubules. In the presented photograph, congestion was observed around the glomerulus and tubules. Congestion and necrosis were observed in most of the kidneys, which are not presented.

The susceptibility of the isolated *P. shigelloides* to antibiotics was determined by disc diffusion [11]. The bacterium was cultivated in a Mueller–Hinton (MH; Difco, Detroit, MI, USA) broth, and the turbidity of the suspension was adjusted to 0.5 McFarland standard (1.5 × 10^8^/mL). The bacterial cultures were subsequently inoculated onto MH agar plates. The concentrations of antibiotics on the disks (BBL; Becton Dickinson, Sparks, MD, USA) were as follows: ampicillin (10 µg), amikacin (30 µg), bacitracin (10 µg), cephalothin (30 µg), chloramphenicol (30 µg), ciprofloxacin (5 µg), cefazolin (30 µg), colistin (10 µg), erythromycin (15 µg), gentamicin (10 µg), kanamycin (30 µg), novobiocin (30 µg), enrofloxacin (5 µg), norfloxacin (10 µg), penicillin (10 µg), streptomycin (10 µg), trimethoprim-sulfamethoxazole (25 µg), oxytetracycline (30 µg), and vancomycin (30 µg). Resistance breakpoints were defined by the National Committee for Clinical Laboratory Standards for gram-negative bacteria [11]. The results of the antimicrobial susceptibility test showed that the bacteria were sensitive to amikacin, cephalothin, chloramphenicol, ciprofloxacin, cefazolin, colistin, gentamicin, kanamycin, enrofloxacin, norfloxacin, streptomycin, and trimethoprim-sulfamethoxazole. The bacteria were resistant to ampicillin, bacitracin, novobiocin, penicillin, oxytetracycline, and vancomycin. The response to erythromycin was intermediate.

### 2.2. Molecular Analysis

For the molecular diagnosis, a genomic DNA sample was prepared from pure cultured colonies from the liver parenchyma and hemorrhagic ascites using a DNeasy^®^ Blood & Tissue kit (Qiagen, Hilden, Germany). PCR was performed using the AccuPower^®^ HotStart PCR premix (Bioneer, Daejeon, Korea) to amplify a variable region of the 23S rRNA gene, in accordance with a protocol from a previous study [12]. PCR products were sent to Solgent (Daejeon, Korea) for bidirectional sequencing. Subsequently, 628 bp 23S rRNA gene fragments were obtained from the liver parenchyma and hemorrhagic ascites.

The sequences were aligned and analyzed using the multiple sequence alignment program CLUSTAL Omega (v. 1.2.1; Belfield, Ireland), and the alignment was corrected using BioEdit (v. 7.2.5; Raleigh, NC, USA). A phylogenetic analysis was performed using MEGA (v. 6.0; State College, PA, USA) based on the maximum likelihood method employing the Kimura two-parameter distance model. The aligned sequences were analyzed using a similarity matrix. The stability of the trees obtained was estimated by a bootstrap analysis for 1000 replicates.

**Figure 3:**
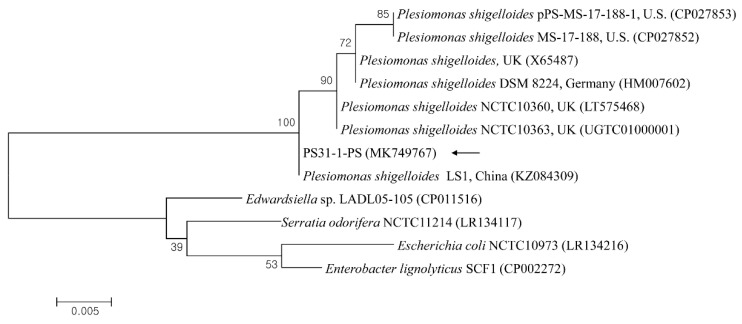
Phylogenetic tree constructed using the maximum likelihood method based on 23S rRNA nucleotide sequences of *Plesiomonas shigelloides*. The black arrow indicates the sequence analyzed in this study. The GenBank accession numbers of other sequences are shown with sequence names. The branch numbers indicate bootstrap support (1000 replicates). The scale bar indicates the phylogenetic distance.

The 23S rRNA sequences of *P. shigelloides* obtained from the liver parenchyma and hemorrhagic ascites were 100% identified, and one sequence (PS31-1-PS) was deposited in the GenBank database (accession no. MK749767). A phylogenetic analysis showed that this sequence (Figure 3) was clustered with previously deposited *P. shigelloides* sequences and shared 98.8–100% genetic identity with several *P. shigelloides* sequences from *Percocypris pingi* in China (100%, KZ084309), the UK (99.7%, LT575468; 99.0%, X65487), Germany (99.5%, HM007602), and the USA (98.8%, CP027853).

## 3. Discussion

The *P. shigelloides* sequence in this study showed a complete concordance with the sequence region determined previously for *P. shigelloides* 23S rRNA gene.

*Plesiomonas shigelloides* has been isolated from various animal species [2], and several documentations of *P. shigelloides*-infection-related diseases have been reported, such as erosive muscle disease in grass carps, epizootic disease in rainbow trout, chronic inflammation and abscess in a lizard, and septicemia in penguins [3,9]. A survey of *P. shigelloides* showed a 3.8% and 10.3% isolation rate in dogs and cats, respectively [1]. Another study found a 0.3% prevalence in zoos [9]. Most animals were asymptomatic, and interestingly, the isolates were the same serovars as those detected in human patients with diarrhea [1], suggesting the possible involvement of human infection [8]. The outbreak in these instances was linked to stress factors, such as overcrowding, oxygen levels, temperature, other climate conditions, and food resources [1,3,9].

In human cases, *P. shigelloides* infections are divided into two groups based on location: gastrointestinal and extraintestinal [5,6,13]. Gastrointestinal *P. shigelloides* are reported in many countries and present as traveler’s diarrhea [8]. Several reports also discuss the extraintestinal form of *P. shigelloides*. Most cases involve septicemia in immunocompromised patients, cellulitis, meningoencephalitis, septic abortion, or septic shock [13,14]. *P. shigelloides* infection is rare, but there are known predisposing factors, such as the consumption of raw or undercooked shellfish, foreign travel, and immunosuppression [1].

Transmission by humans is possible anywhere, and it is thought that the causative agent is everywhere.

However, due to the nature of the bacteria, it is mainly found in water-related places. In this case, it was hypothesized to be caused by contamination with feed rather than by human-to-human transmissions.

In this case, the gray wolf was housed in captivity in an enclosed zoo facility for 7 years. Gastrointestinal infection with *P. shigelloides* may be attributable to the consumption of contaminated food. Among the factors presented in the case history, the deceased gray wolf had a habit of hiding chicken in the ground and consuming it later, a feeding habit that is an important route of infection. Therefore, it was presumed that the number of bacteria was increased by food contamination with *Plesiomonas*, leading to death due to acute bacterial septicemia. The reason was that *P. shigelloides* was isolated from the major organs (heart, liver, kidneys, spleen, and lungs) and blood ascites, and no other bacteria were observed. In addition, the cause of death was acute sepsis caused by infection with *Plesiomonas shigelloides*. The reason was that *Plesiomonas shigelloides* was isolated from major organs, heart, liver, kidney, spleen, lung, and blood ascites, and no other bacteria were observed.

*Plesiomonas* was observed in the stomach contents and ingested feed. While *Escherichia coli* was confirmed in the intestines, it was excluded from the potential cause of the animal’s death because *E. coli* was not isolated from major organs such as liver, kidney, heart, and spleen.

If an oral infection is suspected after the death of an animal, it should be observed in the intestines. Since the bacteria were observed in the liver, a systemic bacterial infection was suspected. Therefore, by looking at bacterial observation in the liver, septicemia can be suspected to be caused by bacteremia. Based on the histological and gross necropsy diagnoses, septicemia can be suspected without the possibility of a passive antemortem infection. A swollen stomach with congestion was very typical of gastric dilation and volvulus, but there was no gastrointestinal organ torsion [15]. While the septicemia caused by *P. shigelloides* was debatable [1,15], there was an outbreak in penguins in a zoo, which was linked to stress brought on by multiple factors [1,3]. Likewise, in gray wolves, stress caused by exhibition and captivity can decrease their immune system function. This may also have contributed to the development of the infection. As seen in animals living in confined spaces, such as zoos, it was thought that the death was due to a complex combination of various factors, such as spatial constraints and visitors.

In captive zoo animals, increased bacterial growth may alter the environment that promotes infections under stress [9,10]. Stress hormones, such as epinephrine and norepinephrine, increase the growth and virulence of bacteria. The small intestine is richly innervated with noradrenergic nerve fibers [10,16]. The secretion of norepinephrine by sympathetic nerve fibers increases under stressful conditions, and this response can affect the susceptibility to diseases [16]. Therefore, the alleviation of captivity-induced stress in zoo animals through the provision of a more relaxing environment, and the minimization of the exposure to stressors might reduce bacterial infections [17].

## 4. Conclusions

This study describes a rare case of systemic gastrointestinal infection due to *P. shigelloides*. To our knowledge, it is the first reported case of this disease in a zoo animal. Zoos should endeavor to decrease the stress levels in animals’ environments, which may affect the mortality rates due to opportunistic infections.

## Data Availability

All the data reported by this case study are contained within the article.
